# ROLE OF POLYMORPHISMS IN VEGF AND KDR GENES IN OSTEOSARCOMA SUSCEPTIBILITY: A SYSTEMATIC REVIEW

**DOI:** 10.1590/1413-785220253302e291664

**Published:** 2025-10-13

**Authors:** AMANDA DOS SANTOS CAVALCANTI, JÉSSICA VILARINHO CARDOSO, VERÔNICA ARAN PONTE, JADE PIRES NASCIMENTO, MARIANA CHANTRE-JUSTINO, ANA CRISTINA DE SÁ LOPES, WALTER MEOHAS, JAMILA ALESSANDRA PERINI

**Affiliations:** 1. Instituto Nacional de Traumatologia e Ortopedia (INTO), Divisao de Pesquisa, Rio de Janeiro, RJ, Brazil.; 2. Universidade do Estado do Rio de Janeiro (UERJ), Laboratorio de Pesquisa em Ciencias Farmaceuticas (LAPESF), Rio de Janeiro, RJ, Brazil.; 3. Instituto Estadual do Cerebro Paulo Niemeyer, Laboratorio de Biomedicina do Cerebro, Rio de Janeiro, RJ, Brazil.

**Keywords:** Osteosarcoma, Genetic Polymorphism, Angiogenesis, VEGF, Systematic Review, Osteossarcoma, Polimorfismo Genético, Angiogênese, Fator de Crescimento Endotelial Vascular, Revisão Sistemática

## Abstract

Osteosarcoma is the most common aggressive primary bone tumor in children and adolescents. Angiogenesis, induced by vascular endothelial growth factor (VEGF) and its receptor (kinase insert domain receptor - KDR) is involved in tumor development. Both genes (VEGF and KDR) are polymorphic, and their association with osteosarcoma remains unclear. A systematic review of observational studies was performed to evaluate the association between polymorphisms in these genes and osteosarcoma development. Pubmed, Medline, Lilacs, and Scielo databases were searched for observational studies published up to April 2024. Eight publications of case-control studies were included, with quality ranging from 82% to 95%. All subjects were from the Chinese population: 1,681 cases and 2,049 controls. A total of six VEGF polymorphisms were analyzed. For osteosarcoma susceptibility, three studies found an increased risk with VEGF rs699947, four with VEGF rs2010963, two with VEGF rs3025039, one with VEGF rs833061, and no studies found an association with the VEGF rs1570360 and VEGF rs10434 SNPs. In addition, no studies evaluated SNPs in the KDR gene and osteosarcoma susceptibility. Further studies in diverse populations, particularly in Brazil, are necessary to clarify the role of VEGF and KDR polymorphisms in osteosarcoma development and prognosis. Level of evidence III; Systematic Review.

## RESUMO

O osteossarcoma é o tumor ósseo primário agressivo mais comum em crianças e adolescentes. A angiogênese, induzida pelo fator de crescimento endotelial vascular (VEGF) e seu receptor domínio quinase (KDR), está envolvida no desenvolvimento tumoral. Ambos os genes (VEGF e KDR) são polimórficos, e sua associação com o osteossarcoma permanece incerta. Uma revisão sistemática de estudos observacionais foi realizada para avaliar a associação de polimorfismos nesses genes com o desenvolvimento de osteossarcoma. As bases de dados Pubmed, Medline, Lilacs e Scielo foram consultadas em busca de estudos observacionais publicados até abril de 2024. Foram incluídas oito publicações de estudos de caso-controle, com qualidade variando de 82% a 95%. Todos os participantes eram da população chinesa: 1.681 casos e 2.049 controles. Foram analisados seis polimorfismos do VEGF. Três estudos encontraram um risco aumentado de desenvolvimento do osteossarcoma com VEGF rs699947, quatro com VEGF rs2010963, dois com VEGF rs3025039, um com VEGF rs833061, e nenhum estudo encontrou associação com os SNPs VEGF rs1570360 e VEGF rs10434. Nenhum estudo avaliou SNPs no gene KDR. Estudos adicionais em populações diversas, especialmente no Brasil, são necessários para esclarecer o papel dos polimorfismos nos genes VEGF e KDR no desenvolvimento e também no prognóstico do osteossarcoma. Nível de Evidência III; Revisão Sistemática.

## INTRODUCTION

Osteosarcoma is a rare malignant primary bone tumor that usually affects long bones such as the distal femur, tibia, and proximal humerus.^1,2^ Its age distribution is bimodal, with a higher peak between 10 and 20 years of age and a second peak between 70 and 80 years of age.^1^ In childhood, the incidence is higher in females, but between 15 and 19 years of age, it becomes higher in males.^3^ The most common symptoms of osteosarcoma are pain and local swelling. The bone becomes fragile due to the tumor presence,^4^ and approximately 17% of patients present with pathologic fractures.^5^ Imaging and laboratory tests aid in the diagnostic investigation; however, the definitive diagnosis is confirmed by histologic evaluation.^6^ The standard treatment consists of neoadjuvant and adjuvant chemotherapy combined with surgery.^7^ Survival rates for patients with localized disease range from 60-75%.^8-10^ On average, 20% of patients have pulmonary metastases at diagnosis.^11^ The presence of metastases significantly decreases overall survival in 10 years, approximately 12.5% in Brazil^11^ and 25% in the United States and Europe.^12^

The mechanism of osteosarcoma development is complex, and its exact etiology is still unknown. However, environmental and genetic factors have been associated with the development of this type of tumor.^13^ Additionally, angiogenesis is a crucial process in the development, and proliferation of tumor cells, cancer progression, and metastasis development.^14^ Vascular endothelial growth factor (VEGF) is one of the main pro-angiogenic factors, and high expression of VEGF and its kinase insert domain receptor (KDR) has been observed in osteosarcoma tissue samples compared to adjacent tissues.^15,16^ VEGF expression was significantly correlated with histological grade, tumor stage, distant metastasis, and lower survival.^17-19^ Positive VEGF status in tumor samples from osteosarcoma patients negatively impacts overall survival and disease-free survival, suggesting this factor is an effective biological marker for prognosis.^20,21^ In this regard, anti-angiogenic inhibitor drugs targeting VEGF/VEGFR successfully inhibited osteosarcoma angiogenesis and cell proliferation *in vivo* and *in vitro,* ^22^ supporting the relevance of VEGF and its receptor in osteosarcoma.

The *KDR* and *VEGF* genes are located on chromosomes 4q11-q12 and 6p21.1, respectively, and have several single nucleotide polymorphisms (SNPs) in the coding and regulatory regions that may affect enzyme activity or expression.^23-25^ In addition, different populations show increased frequencies of these SNPs.^26-28^ Additionally, *VEGF* SNPs have been associated with osteosarcoma risk in the Chinese population^29-34^ although the literature reports show controversial results. Different isoforms of VEGF have been associated with osteosarcoma progression,^35^ metastases development,^36^ and poor response to chemotherapy.^37^ Among these, the most important isoform involved in angiogenesis and vasculogenesis is VEGF-A,^38^ which is the subject of the current study. In this context, the aim of this study was to perform a systematic review of observational studies evaluating *VEGF* and *KDR* SNPs and osteosarcoma susceptibility to discuss the causes of the previously described conflicting results.

## METHODS

### Systematic Review Search Strategy

A literature search was conducted in the Pubmed, Medline, Lilacs, and Scielo databases to identify all articles that evaluated SNPs in the VEGF and KDR genes associated with the risk of developing osteosarcoma. These last two databases were consulted with specific interest in studies involving Latin American populations. All studies published up to April 2024 were evaluated, considering the following combined descriptors in both English and Portuguese: osteosarcoma and (“polymorphism” or “SNP” or “genetic polymorphism”) and (“VEGF” or “Vascular endothelial growth factor” or “VEGFR-2” or “Vascular endothelial growth factor-2” or “KDR” or “Kinase Insert Domain Receptor”). Additionally, the reference list of review/meta-analysis articles and of each selected article were reviewed to identify any other articles that might not have been included in the initial data search.

### Inclusion and Exclusion Criteria

The articles selected for this review followed the following inclusion criteria: (i) observational studies that evaluated the association of SNPs in the VEGF and KDR genes with the development of osteosarcoma; (ii) studies published in English or Portuguese; (iii) studies published up to April 2024. The exclusion criteria were: (i) publications that did not include osteosarcoma patients, (ii) did not analyze VEGF and KDR SNPs; (iii) analyzed only disease progression; and (iv) publications without completely accessible texts.

### Data Extraction

After selecting the studies according to the inclusion and exclusion criteria, the following information was extracted from each article by two reviewers (JVC and JPN): first author and year of publication; study population; the number of osteosarcoma cases and controls; the age of both groups; gender; type of control recruited; patient-reported family history of cancer; location of the osteosarcoma; presence or absence of metastasis; clinical staging of the osteosarcoma cases; identification of SNPs; genotyping technique for SNP identification; SNP allele frequency data; and Pearson’s chi-square test and odds ratio (OR) values with their respective 95% confidence intervals (CI 95%).

### Data management

The data extracted from the studies included in this review were organized and analyzed using Excel spreadsheets. Percentages were used as statistical measures to compare the frequency of each variable extracted from the studies, followed by a descriptive analysis of the data. To aid in data synthesis and visualization, Excel tools were employed for basic calculations such as sums and percentages, as well as for arranging the characteristics of each study. All data were reviewed by two researchers (JVC and JPN). When an article did not report the frequency of the alleles for the polymorphisms included, the frequencies were calculated based on genotype information. Since each individual has two alleles, the total number of alleles was determined by summing the set of genotypes.

### Quality Assessment of Included Studies

All included studies were independently analyzed by two reviewers (JVC and JPN) according to the STROBE checklist used for observational studies. This quality assessment tool has twenty-two items related to: title and abstract (item 1), introduction (items 2 and 3), methodology (items 4 to 12), results (items 13 to 17), discussion (items 18 to 21), and funding of each study (item 22), assigning a score of 0 or 1 for each item. In the end, studies with a score greater than 50% were approved for evaluation in this review according to the guideline.^39^

## RESULTS

**Figure 1:**
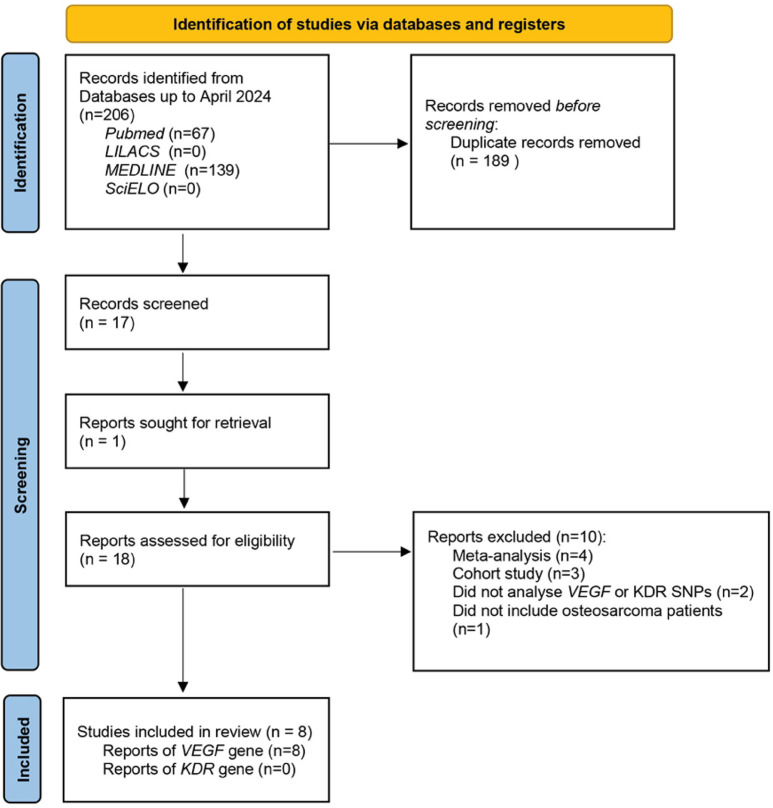
Study flowchart.

The flowchart of the included articles is illustrated in Figure 1. Out of the 206 publications found, we excluded 189 duplicates and were left with 17 valid publications. One additional article^31^ was identified after reviewing the reference list of the 17 selected articles, making a total of 18 articles for evaluation of eligibility criteria. Subsequently, 10 articles were excluded: 4 meta-analyses,^40-43^ 3 studies that analyzed only disease progression,^16,44,45^ 2 studies that did not analyze SNPs in the *VEGF* or *KDR* genes,^46,47^ and one study that did not include osteosarcoma patients.^48^ Consequently, eight publications^29-34,49,50^ were selected for full-text evaluation and included in this review. All eight studies investigated SNPs in the VEGF gene only. No publication was found that described an association of SNPs in the KDR gene with osteosarcoma development (Figure 1).

Table 1 describes the characteristics of osteosarcoma cases from the studies included in this review. Regarding the STROBE rating of the included articles, the studies had a quality score of over 80%, with Wang et al.^50^ having the highest score (21 out of 22 items) and the studies by Hu et al.^29^ Zhang et al.^33^ and Zhang et al.^34^ having the lowest scores (18 out of 22 items).

**Table 1:** Demographic and clinical data of osteosarcoma cases from eligible studies and quality assessment of included articles.

Reference	Population (n)*	Age (%)		Sex (%)	Family history of cancer (%)	Topography (%)		Metastasis (%)	Tumor Staging (%)		Strobe (%) #
		≤ 20	> 20	Men	Yes	Long bones	Axial skeleton	Yes	I-II	III-IV	
Tie et al.^32^	Chinese (165)	92 (55.6)	73 (44.4)	108 (65.3)	145 (87.6)	118 (71.3)	47 (28.7)	37 (22.3)	105 (63.4)	60 (36.6)	19 (86)
Wang et al.^50^	Chinese (330)	251 (76.1)	79 (23.9)	188 (57.0)	49 (14.8)	257 (78.9)**	73 (21.1)**	87 (26.4)	NI	NI	21 (95)
Hu et al.^29^	Chinese (130)	80 (61.5)	50 (38.5)	76 (58.5)	NI	94 (72.3)	36 (27.7)	28 (21.5)	89 (68.5)	41 (31.5)	18 (82)
Li-Lian et al.^30^	Chinese (176)	81 (46.0)	95 (54.0)	109 (61.9)	160 (90.9)	134 (76.1)	42 (23.9)	NI	101 (57.4)	75 (42.6)	19 (86)
Liu et al.^31^***	Chinese (186)	119 (64.0)	67 (36.0)	114 (61.3)	NI	135 (72.6)	51 (27.4)	143 (76.9)	NI	NI	20 (91)
Zhang G. et al.^33^	Chinese (180)	123 (68.3)	57 (31.7)	110 (61.1)	163 (90.6)	128 (71.1)	52 (28.9)	35 (19.4)	105 (58.3)	75 (41.7)	18 (82)
Zhang H. F. et al.^34^****	Chinese (182)	82 (45.1)	100 (54.9)	106 (58.2)	167 (91.8)	125 (68.7)	57 (31.3)	63 (34.6)	NI	NI	18 (82)
Cao et al.^49^	Chinese (322)	217 (67.4)	105 (32.6)	231 (71.7)	24 (7.5)	226 (70.2)	96 (29.8)	80 (24.8)	NI	NI	20 (91)

*Number of osteosarcoma cases in each article. **Tumor location categorized as extremities (long tubular bones) or other (axial skeleton).^50^ ***Reported collecting information on tumor staging, family history of cancer, and histological type (osteoblastic, chondroblastic, fibroblastic, and mixed types); however, the sample size of each was not described.^31^ ****Reported collecting information on tumor stage, but the sample size for each was not described.^34^ #22 items related to the STROBE checklist. NI – not informed.

The study population in all articles was Chinese, with a total of 1,681 osteosarcoma cases analyzed, with the majority (75%) of articles presenting more individuals under the age of 20 years,^29,31-33,49,50^ and males being the most common sex in all articles (100%). Only the studies by Hu et al.^29^ and Liu et al.^31^ did not include information on family history of cancer (25%). Among the other studies, four (50%) observed a high frequency of cancer in the family^30,32-34^ and two (25%) reported a low frequency of this characteristic.^49,50^ In all articles, osteosarcoma was more frequently located in the long bones, and only in the article by Liu et al.^31^ about 77% of patients had metastasis, while the other studies (n = 6; 75%) observed a much lower frequency (ranging from 19.4 to 34.6%).^29,32-34,40,50^ The study by Li-Lian et al.^30^ did not include this information. In addition, four (50%) studies^29,30,32,33^ reported stage I-II being the most common. Wang et al.^50^ Liu et al.^31^ Zhang et al.^34^ and Cao et al.^49^ did not report the tumor staging, although Liu et al.^31^ reported collecting this information along with histologic subtype (osteoblastic, chondroblastic, fibroblastic, and mixed types). However, the frequency of both characteristics was not found in the text.

The demographic characteristics and inclusion and exclusion criteria of the control participants from the selected studies (a total of 2,049 controls) are also described in this review (Table 2). The study period of the included articles ranged from 2008 to 2015, with seven being hospital-based (87.5%) and one being population-based.^50^ For controls (healthy individuals), most studies (n = 7; 87.5%) reported a negative personal history of cancer and matched controls to cases by age and sex. In the article by Cao et al.^49^ the authors only reported a negative personal history of cancer for healthy individuals. In the study by Wang et al.^50^ controls were also matched by area of residence. Li-Lian et al.^30^ and Zhang et al.^34^ also reported a negative personal history of chronic diseases, while only Liu et al.^31^ reported that the controls had no family history of cancer. In most articles (75%), the controls were under 20 years of age,^9,31-34,49,50^ with a higher frequency of males observed in all studies. Four studies (50%) reported a high frequency of family history of cancer in the control group.^30,32-34^ This characteristic was less frequent only in the studies of Wang et al.^50^ and Cao et al.^49^ Hu et al.^29^ did not provide this information.

**Table 2:** Demographic characteristics and criteria used to select the controls from eligible studies.

Reference	Chinese-Region (n)*	Study Period	Study Design	Type of Control	Control matched by	Age (%)		Sex (%)	Family history of cancer (%)
						≤ 20	> 20	Men	Yes
Tie et al.^32^	Inner Mongolia (330)	2011 to 2013	HB	RE, NegCanP	Sex and age	170 (51.6)	160 (48.4)	215 (65.3)	303 (91.8)
Wang et al.^50^	Ningxia (342)	2009 to 2013	PB	RE, NegCanP	Sex, age, and residence	244 (71.3)	98 (28.7)	176 (51.5)	33 (9.6)
Hu et al.^29^	Wuhan (130)	2011 to 2013	HB	RE, NegCanP	Sex and age	77 (59.2)	53 (40.8)	76 (58.5)	NI
Li-Lian et al.^30^	Beijing (176)	2011 to 2013	HB	RE, NegCanP, NegChr	Sex and age	86 (48.9)	90 (51.1)	109 (61.9)	163 (92.6)
Liu et al.^31^	Jinan (186)	2008 to 2010	HB	RE, NegCanP, NegCanF	Sex and age	117 (62.9)	69 (37.1)	114 (61.3)	NA
Zhang G. et al.^33^	Chongqing, Inner Mongolia (360)	2011 to 2013	HB	RE, NegCanP	Sex and age	236 (65.6)	124 (34.4)	220 (61.1)	349 (96.9)
Zhang H. et al.^34^	Weifang (182)	2011 to 2013	HB	RE, NegCanP, NegChr	Sex and age	86 (47.2)	96 (52.8)	106 (58.2)	171 (94.0)
Cao et al.^49^	Shanghai (343)	2009 to 2015	HB	NegCanP	NI	217 (67.4)	130 (37.9)	242 (70.6)	27 (7.9)

*Number of controls in each article. HB = hospital-based. PB = population-based. RE = routine examination. NegCanP = negative personal history of cancer. NegCanF = negative family history of cancer. NegChr = negative history of chronic diseases. NA = not applicable once controls did not have a family history of cancer. NI = not informed by authors.

**Figure 2:**
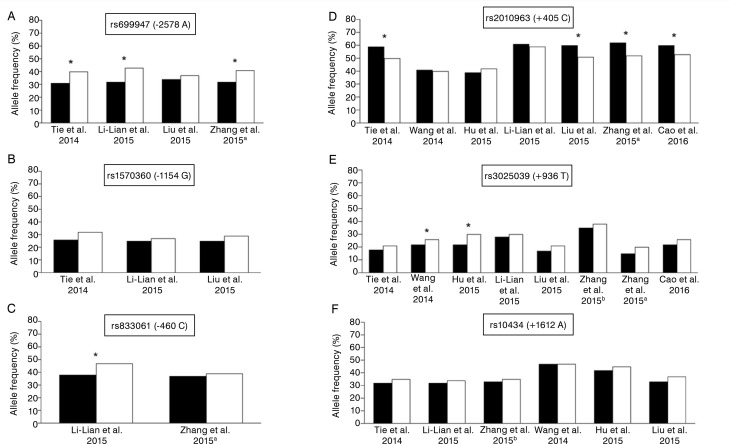
Frequency of variant alleles of the SNPs *VEGF* rs699947 *A* (A), *VEGF* rs1570360 *G* (B), *VEGF* rs833061 *C* (C), *VEGF* rs2010963 *C* (D), *VEGF* rs3025039 *T* (E), and *VEGF* rs10434 *A* (F) found in the studies included in this review. Zhang H. et al. 2015;^34^ Zhang G. et al, 2015.^33^ * P-value of Person's Chi-square test < 0.05.

Figure 2 displays the frequencies of the variant alleles of each *VEGF* gene SNP found in the eligible studies of this systematic review. For the rs699947 *C>A* SNP, the variant allele frequency ranged from 37.4% to 42.6% in the case group and from 31.2% to 33.6% in the control group. Of the four published articles on this SNP,^30-32,34^ three^30,32,34^ found a positive association with the development of osteosarcoma (Figure 2A; Table 3). For the rs1570360 *A>G* SNP, the variant allele frequency ranged from 26.8% to 31.7% in cases and from 24.5% to 26.2% in controls, with no significant differences observed between groups observed in any study (Figure 2B; Table 3).^30-32^ Regarding the rs833061 *T>C* SNP, the variant allele frequency ranged from 39.3% to 47.4% in cases and from 36.8% to 38.3% in controls, with only Li-Lian et al.^30^ finding an increased risk of developing osteosarcoma with the CC genotype or the dominant model (TC+CC) (Figure 2C; Table 3). Seven articles evaluated the rs2010963 *G>C* SNP,^29-33,49,50^ with four reporting a positive association with disease development in the presence of the G allele or GG genotype.^31-33,49^ The variant allele frequency varied from 40.2% to 58.8% in the case group and from 39.1% to 61.8% in the control group across published studies (Figure 2D; Table 3).

**Table 3:** Description of the results regarding the association of the studied polymorphisms in the *VEGF* gene with osteosarcoma.

SNP (rs)	Localization*	Genotypes	OR (CI 95%)	Reference
rs699947	-2578C>A (PR)	CA and AA	No association	Liu et al.^31^
		AA CA+AA	2.3 (1.2-4.6) 1.7 (1.1-2.6)	Li-Lian et al.^30^
		AA CA+AA	2.0 (1.02-3.8) 1.6 (1.01-2.4)	Zhang H. et al.^34^
		AA	2.06 (1.1-3.5)	Tie et al.^32^
rs1570360	-1156 (or -1154) A>G (PR)	AG and GG	No association	Liu et al.^31^
		AG, GG, and AG+GG	No association	Li-Lian et al.^30^
		AG and GG	No association	Tie et al.^32^
rs833061	-460T>C (PR)	CC TC+CC	2.2 (1.1-4.2) 1.6 (1.0-2.6)	Li-Lian et al.^30^
		TC, CC, and TC+CC	No association	Zhang H. et al.^34^
rs2010963	-634 (or +405) G>C (5'UTR)	GG	2.0 (1.1-3.8)	Liu et al.^31^
		GC, CC, and GC+CC	No association	Hu et al.^29^
		CG, GG, and CG+GG	No association	Li-Lian et al.^30^
		GG	2.1 (1.2-3.7)	Tie et al.^32^
		GC, CC, and GC+CC	No association	Wang et al.^50^
		GG	2.3 (1.3-4.0)	Zhang G. et al.^33^
		GG	1.5 (1.04-2.3)	Cao et al.^49^
rs3025039	+936C>T (3'UTR)	TT	2.7 (1.02-8.3)	Hu et al.^29^
		CT and TT	No association	Liu et al.^31^
		CT, TT, and CT+TT	No association	Li-Lian et al.^30^
		CT and TT	No association	Zhang G. et al.^33^
		CT, TT, and CT+TT	No association	Zhang H. et al.^34^
		CT and TT	No association	Tie et al.^32^
		TT	2.7 (1.3-5.5)	Wang et al.^50^
		TT and CT+TT	No association	Cao et al.^49^
rs10434	+1612G>A (3'UTR)	GA, AA, and GA+AA	No association	Hu et al.^29^
		GA and AA	No association	Liu et al.^31^
		CT, TT, and CT+TT	No association	Li-Lian et al.^30^
		CT and TT	No association	Zhang G. et al.^33^
		CT and TT	No association	Tie et al.^32^
		GA, AA, and GA+AA	No association	Wang et al.^50^

SNP = single nucleotide polymorphism; *Position and region of the polymorphism in the gene: PR = promoter region; UTR = untranslated region. OR = odds ratio. CI = confidence interval. (+) = dominant models.

All articles in this review examined the rs3025039 C>T SNP, but only Wang et al.^50^ and Hu et al.^29^ observed an approximately twofold increased risk of developing osteosarcoma. The frequency of the rs3025039 T variant allele ranged from 20.1% to 37.8% in cases and from 15.4% to 34.6% in controls (Figure 2E; Table 3). Finally, six articles examined the rs10434 G>A SNP,^29-33,50^ and none of them found a significant association with tumor development. The frequency of the variant allele ranged from 33.5% to 47.4% in cases and from 32.2% to 46.5% in controls (Figure 2F; Table 3). It is worth noting that, of the articles included in this review, only one evaluated both the impact of polymorphisms on the risk of developing osteosarcoma and their prognostic value. The authors^31^ associated the polymorphism rs2010963 (-634GG) with a shorter overall survival (OS) time (HR, 3.10; 95% CI, 1.17-8.38).

Table 3 details the characteristics of the *VEGF* gene SNPs and the data from the association analyses of each study. The eight studies included in this review, identified six VEGF gene variants: rs699947 *C>A,* rs1570360 *A>G,* rs833061 *T>C,* rs2010963 *G>C,* rs3025039 *C>T*, and rs10434 *G>A*. The first three variants are located in the promoter region, one in the 5’ UTR, and the remaining two in the 3’ UTR, respectively. All studies used the same genotyping technique for these SNPs, polymerase chain reaction-restriction fragment length polymorphism (PCR-RFLP).

Additionally, Li-Lian et al.^30^ Tie et al.^32^ and Zhang G. et al.^33^ performed stratification analyses based on environmental characteristics for the SNPs in their respective studies. Li-Lian et al.^30^ found no associations for any variables, whereas Tie et al.^32^ reported that the rs699947 *AA* and rs2010963 *GG* genotypes were associated with an increased risk of osteosarcoma in individuals younger than 20 years and those with a family history of cancer. Furthermore, the rs699947 and rs2010963 genotypes were associated with an increased risk of developing osteosarcoma in males and females, respectively. Zhang et al.^33^ also found that the rs2010963 GG genotype was associated with an increased risk in individuals younger than 20 years, in both sexes and with a family history of cancer. In addition, Liu et al.^31^ evaluated the survival of osteosarcoma cases and found that the rs2010963 SNP could be considered as a prognostic factor for the disease, with the rs2010963 genotype being associated with a threefold higher risk of decreased survival (OR = 3.1; 95% CI = 1.17-8.38).

## DISCUSSION

This study conducted a systematic review of observational studies investigating the potential relationship between SNPs in the *VEGF* and *KDR* genes and the risk of developing osteosarcoma. However, at the time of the literature review, none of the published studies had evaluated the association between SNPs in the KDR gene and osteosarcoma susceptibility. Eight studies examined the interaction of *VEGF* SNPs and osteosarcoma susceptibility,^29-34,49,50^ and only four SNPs (rs699947, rs833061, rs2010963, and rs3025039) were associated with osteosarcoma risk.^29-34,49,50^ Furthermore, one *VEGF* SNP (rs2010963) was associated with shorter survival.^31^

In most articles, osteosarcoma cases involved men under the age of 20, which is consistent with other studies conducted in different populations.^1,51^ A study by our group of Brazilian patients with osteosarcoma recruited from a reference orthopedic hospital included samples from 37 histologically confirmed patients (24 males and 13 females) with a mean age of 18 years,^52^ which is consistent with the current systematic review. A family history of cancer was highly prevalent (87.6-91.8% and 91.8-96.9%) among cases and controls, respectively, in four of the selected studies.^30,32-34^ Various studies have found that a family history of cancer increases the likelihood of developing endometrial and prostate cancer by 3.1% and 59.7%, respectively, and that 15-20% of breast cancer cases are associated with a family history of cancer.^53,54^ For osteosarcoma, a 3.6-fold risk was observed, associated with maternal endocrine gland cancer.^55^

In all reviewed articles, osteosarcoma was more commonly found in long bones, which is consistent with the literature.^1,51^ The growth plate has a higher rate of cell proliferation, making cells more susceptible to mutations.^51^ Similarly, our group’s study showed that most tumors were located in the knee region (81%) and the most common histologic variant was high-grade central conventional osteosarcoma (83.8%), with 27% presenting metastases.^52^ Most patients in this review did not have metastases and had stage I-II tumors, corroborating other studies.^56,57^ Advanced age, axial skeletal location, lesion size, and residence in disadvantaged areas are risk factors associated with the presence of metastases at diagnosis.^56^

The angiogenic process involving VEGF and its receptors plays an important role in bone tissue regeneration and in the growth, progression, and metastasis of bone tumors.^58^ Studies suggest an imbalance between angiogenic inducers and inhibitors, leading to an angiogenic tumor phenotype and tumor development.^15,59^ In osteosarcoma, *VEGF* expression is associated with poor prognosis and metastasis development.^60^ *In vitro*, VEGF silencing inhibits proliferation and promotes apoptosis in SaOS-2 cells, while in vivo, it suppresses tumor growth and angiogenesis.^61^ Zheng et al.^62^ showed higher expression of VEGF receptor (VEGFR2) in metastatic tumor samples (lung) compared to the primary tumor, and receptor inhibition reduces metastatic ability in vitro and significantly attenuates metastatic capacity *in vivo*.

In 2016, a meta-analysis of seven articles to provide a comprehensive assessment of the associations of SNPs in the *VEGF* gene with osteosarcoma susceptibility found that the *VEGF* rs3025039 *C>T* and rs2010963 *G>C* SNPs were associated with increased risk and protection, respectively, and that the rs10434 *G>A* SNP had no association with disease development.^40^ Then, in 2017, another meta-analysis observed that the *VEGF* rs699947 C>A, rs1570360 A>G, rs833061 T>C, rs2010963 G>C, and rs3025039 C>T SNPs were associated with osteosarcoma risk in the Han Chinese population, while the *VEGF* rs10434 G>A SNP showed no significant association with the disease.^43^ Recently, in 2022, another meta-analysis highlighted that the *VEGF* rs1570360 *A>G,* rs2010963 *G>C,* and rs3025039 *C>T* SNPs were associated with osteosarcoma risk, while the C allele of the *VEGF* rs699947 *C>A* SNP had a protective effect.^42^ More recently, a meta-analysis that included the analysis of five SNPs, four of which were also evaluated in this review. The data from Hassanain et al.^63^ support the risk analysis for the *VEGF* rs3025039 TT and rs2010963 GG SNPs. In contrast to our results, the leave-one-out analysis showed that the *VEGF* rs699947, rs10434, and rs1570360 SNPs were not significant. The *VEGF* rs833061 CC SNP was excluded from the meta-analysis because of the low quality of the articles. However, in our review, the association (2-fold risk) was based on the study by Li-Lian et al.^30^ which was not cited in the meta-analysis by Hassanain et al. Similarly, contrary to our findings, the authors reported a risk associated with the *VEGF* rs157090360 based on Zhao’s meta-analysis,^43^ which was not included in this review once we excluded meta-analysis studies.

The *VEGF* rs699947 C>A, rs1570360 A>G, and rs833061 T>C SNPs may affect transcriptional activity and lead to increased protein levels because they are located in the promoter region of the gene.^64^ The *VEGF* rs2010963 G>C SNP, located in the 5’UTR, is significantly positively correlated with VEGF protein synthesis by peripheral blood mononuclear cells (PBMCs).^24^ Meanwhile, the *VEGF* rs3025039 C>T SNP, located in the 3’UTR, may influence plasma VEGF levels.^23^ For *VEGF* rs10434 G>A SNP, also located in the 3’UTR, no studies have yet confirmed its influence on the gene; however, this type of SNP may affect mRNA stability and alter the ability of microRNAs to interact with their target due to its position in the gene.^65^

Although most of the studies included in this review reported similar frequencies of *VEGF* SNPs, they were all conducted in the Chinese population, which could lead to the inconsistencies in the association results. These inconsistencies may also be explained by (i) variability in sample size, which may affect risk estimates, and (ii) the type of control used, which may also be associated with the variants studied. In case-control studies, the selection of the control group is critical, as these individuals should not have the chance of developing the outcome or have risk factors similar to the outcome, such as SNPs in the *VEGF* gene, which could bias risk estimates. Most articles in this review recruited healthy hospital-based controls matched to the case group by age and sex and with a negative personal history of cancer, which is a positive aspect as the ideal control group is directly determined by the definition and selection of the case group and recruited from the same source.^66^ A limitation of the present review, common to other literature reviews, is the possibility that we did not include all the literature and missed some relevant studies. We included three databases, English-language articles only, and did not include grey literature.

Comparing the frequencies of *VEGF* rs699947 *A,* rs1570360 G*,* rs833061 C, and rs2010963 C SNPs in populations from different regions worldwide (according to data from the National Center for Biotechnology Information - NCBI), with the Chinese population (from this review) and the Brazilian population,^67^ which has an extensive admixture of three major ancestral roots (indigenous, European and African) and is predominant in today’s Brazilian population.^68^ For *VEGF* rs699947 A SNP, the frequencies in Chinese (31.2-33.6%), Asian (28-34%) (NCBI), and Brazilian (31.9%) populations^67^ were found to differ from those in African American (23%) and Sub-Saharan African (12-17%) populations (NCBI). For *VEGF* rs1570360 G SNP, the frequencies in European (67-69%), African (91-97%), American (78-80%), Latin American (75-80%) (NCBI), and Brazilian populations (83.8%)^67^ differ from those in the Chinese population (24.5-26.2%). For the *VEGF* rs833061 C SNP, the frequencies in European (47%) (NCBI) and Brazilian (41.7%) populations^67^ differ from those in Asian (27-34%), Chinese (38.3-36.8%), African American (33%), and Sub-Saharan African (30%) populations (NCBI). Finally, for the *VEGF* rs2010963 C and rs3025039 SNPs, the frequencies in European (27-42%; 10-23%, respectively), African American (24-36%; 5-12%, respectively) (NCBI), and Brazilian (38.6% and 12.5%, respectively) populations^67^ differed from those in the Chinese population (39.1-61.8%; 15.4-34.6%, respectively).

The inconsistencies in SNP frequencies reinforce the idea that the variants studied in this review may exist at variable frequencies in both more homogeneous populations, such as the Chinese, and more heterogeneous populations, such as the Brazilians. This phenomenon may occur because of factors such as long-distance migration and the encounter of different populations during demographic expansion, resulting in genetic mixing or complete population replacement.^69,70^ In addition, genetic interactions with environmental factors may influence these frequencies.^71^ Therefore, extrapolation of data from Chinese SNP association studies to mixed populations is likely to lead to conclusions that are inconsistent with reality. In addition, the paucity of studies evaluating SNPs in the *KDR* gene identified in this review highlights the need for further investigation of this gene, given its importance in VEGF signaling and consequently in the angiogenic process.

## CONCLUSIONS

For osteosarcoma susceptibility, three studies found an increased risk with *VEGF* rs699947, four with *VEGF* rs2010963, two with *VEGF* rs3025039, one with *VEGF* rs833061, and no studies found an association with the *VEGF* rs1570360 and *VEGF* rs10434 SNPs. In addition, no studies evaluated SNPs in the *KDR* gene and osteosarcoma susceptibility.

## Figures and Tables

**Figure:**
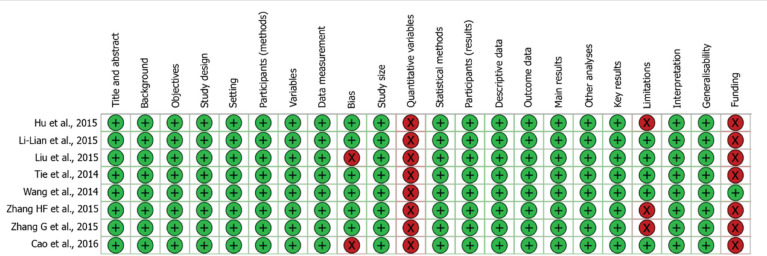
Quality assessment by the STROBE checklist. Prepared using: McGuinness LA, Higgins JPT. Risk-of-bias VISualization (robvis): An R package and Shiny web app for visualizing risk-of-bias assessments. Res Synth Methods. 2021;12(1):55-61.
